# Injectable Vancomycin Contained Within a Hyaluronic Acid-Impregnated Alginate Carrier: A Novel Technique for Prophylaxis Against Surgical Site Infection

**DOI:** 10.7759/cureus.102630

**Published:** 2026-01-30

**Authors:** Calvin J Rushing

**Affiliations:** 1 Foot and Ankle Surgery, Dallas Orthopedic and Shoulder Institute, Dallas, USA

**Keywords:** antibiotics, foot & ankle, fracture, infection, osteoblast, versacoat, versawrap

## Abstract

Topical vancomycin powder is used intraoperatively to mitigate the risk of surgical site infection. The method is purported to create supraphysiologic levels of the antibiotic locally, preventing biofilm formation and bacterial colonization, with only a transient effect systemically. However, the concentration and duration of the effect of antibiotics locally remains unclear; concern(s) regarding potential effects on mesenchymal stem cell osteogenic differentiation have been raised and postoperative maceration is not uncommon. The combination of vancomycin with an impregnated carrier processing a gradual dissolution profile over time could theoretically improve efficacy by minimizing any sudden “high concentration burst” and reducing postoperative drainage while prolonging the physiologic levels locally. The purpose of the present article is to describe the author's novel techniques for injectable vancomycin powder contained within a hyaluronic acid-impregnated alginate carrier for prophylaxis against surgical site infection.

## Introduction

Surgical site infection (SSI) is one of the most common complications after foot and ankle surgery, with an incidence exceeding the rates reported for hand surgery, total shoulder arthroplasty, total hip arthroplasty, and total knee arthroplasty [1-5]. Researchers have attributed the unacceptably high SSI rate to a number of factors and various mitigation strategies have been introduced including preoperative skin antisepsis, surgical irrigants, topically applied vancomycin powder, and postoperative prophylactic antibiotics [6-11]. However, there is a paucity of research on the topic of vancomycin powder in the foot and ankle, and no consensus exists regarding the use of prophylactic antibiotics after foot and ankle surgery [12-15]. 

Vancomycin is a glycopeptide antibiotic that inhibits cell wall formation in Gram-positive bacteria. The application of topical vancomycin powder for SSI prophylaxis has been reported across a number of orthopaedic procedures. Proponents for use in the foot and ankle cite reduced rates of SSI in high-risk patient populations, few adverse reactions, and a nominal cost [9]. Opponents cite concern over purported non-union risk based on an in vitro study, and postoperative wound drainage/maceration as reasons to eschew from use [10].

Currently, the optimal dosage, resultant concentration, and duration of activity after application of topically applied vancomycin for foot and ankle surgery remains to be determined. In an in vitro study, Bariteau et al. concluded that at high concentrations (500 μg/ml, 5000 μg/ml), vancomycin may impair the viability and osteogenic differentiation of human mesenchymal stromal cells (hMSCs) [10]. The authors cautioned against the use of vancomycin powder in fracture- and arthrodesis-type procedures. In contrast, Hernandez et al. showed that clinically relevant doses of vancomycin powder did not impair osteogenesis or fracture healing in a diabetic rodent fracture model [11]. From the referenced studies and available spine research, one may surmise the effect of topically applied vancomycin on hMSCs is both concentration- and time-dependent.

The combination of vancomycin powder with an impregnated carrier processing a gradual dissolution profile over time could therefore theoretically improve efficacy by minimizing any sudden “high concentration burst”, prolonging physiologic levels locally, and potentially reducing postoperative drainage. An opposing charge for the carrier (negative) would be particularly suitable in combination with vancomycin (positive charge). The purpose of the present article is to describe the author's novel techniques for injectable vancomycin powder contained within a hyaluronic acid-impregnated alginate carrier for prophylaxis against SSI.

## Technical report

Technique

The author's original technique, as well as the modified techniques, are described. The original technique required a greater volume of saline for mixing, used a large bore needle for injection, and was more labor-intensive for the surgical scrub technician (10 min). The first modified technique incorporated the help of a circulating nurse and was conceived to improve the process of mixing, mitigate the dilution of the hyaluronic acid-impregnated alginate carrier, and afford injection of a lower volume with an 18-gauge needle. The technique is less labor-intensive for the surgical scrub technician and requires approximately half the time of the original technique (5 min). The second modified technique incorporated a newer product; prepackaged syringe-delivered flowable hydrogel. The concentration of the carrier using the prepackaged flowable hydrogel is more than twice that of the other techniques, which reduces potential for carrier dissolution, and is even less labor-intensive (<4 min).

For the first two techniques, the following are required: (1) vancomycin powder (1 g), (2) sterile saline solution, (3) specimen cup, (4) hemostat or freer elevator, (5) 10 and 20 cc luer lock syringes, and (6) VersaWrap (Alafair Biosciences, Inc. Austin, TX), 5 by 5 cm (one to two sheets). For the most recent technique, the following is required: (1) vancomycin powder (1 g), (2) sterile saline solution, (3) 10 cc luer lock syringe, and (4) Versa-Coat (Alafair Biosciences). 

Original technique

**Figure 1:**
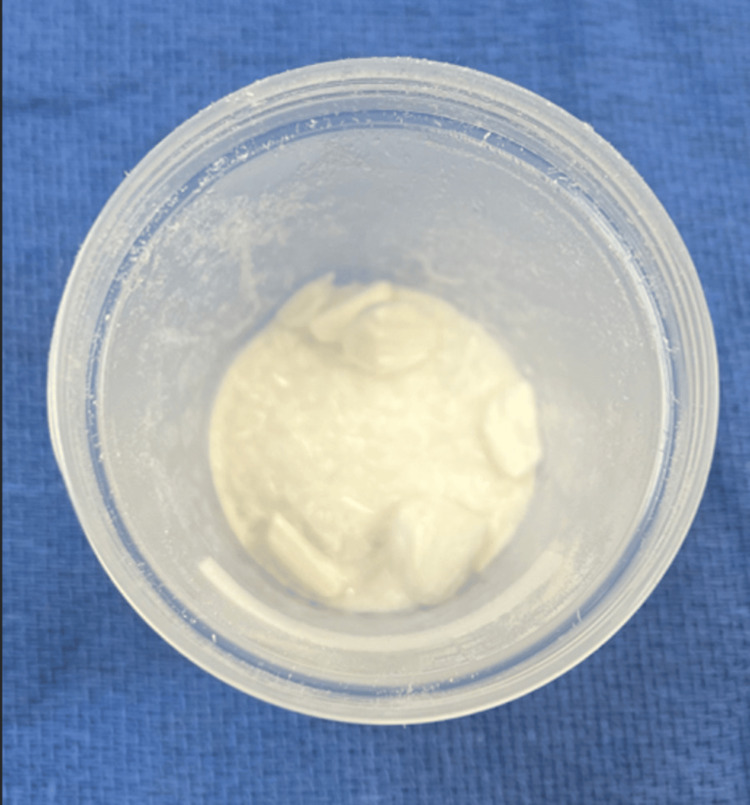
Vancomycin (1 g) powder is placed in a specimen cup.

**Figure 2:**
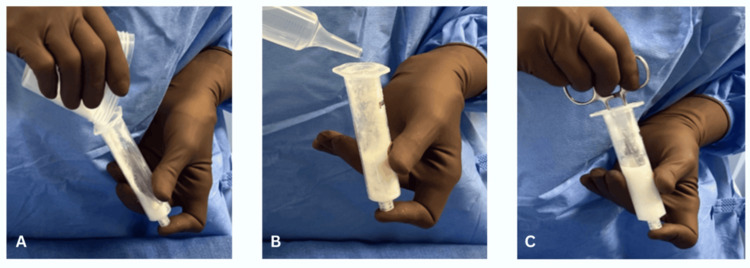
(A) Vancomycin powder is transferred into a 20 cc leur lock syringe, (B) 6-8 cc sterile saline is added, and (C) manual mixing is performed.

**Figure 3:**
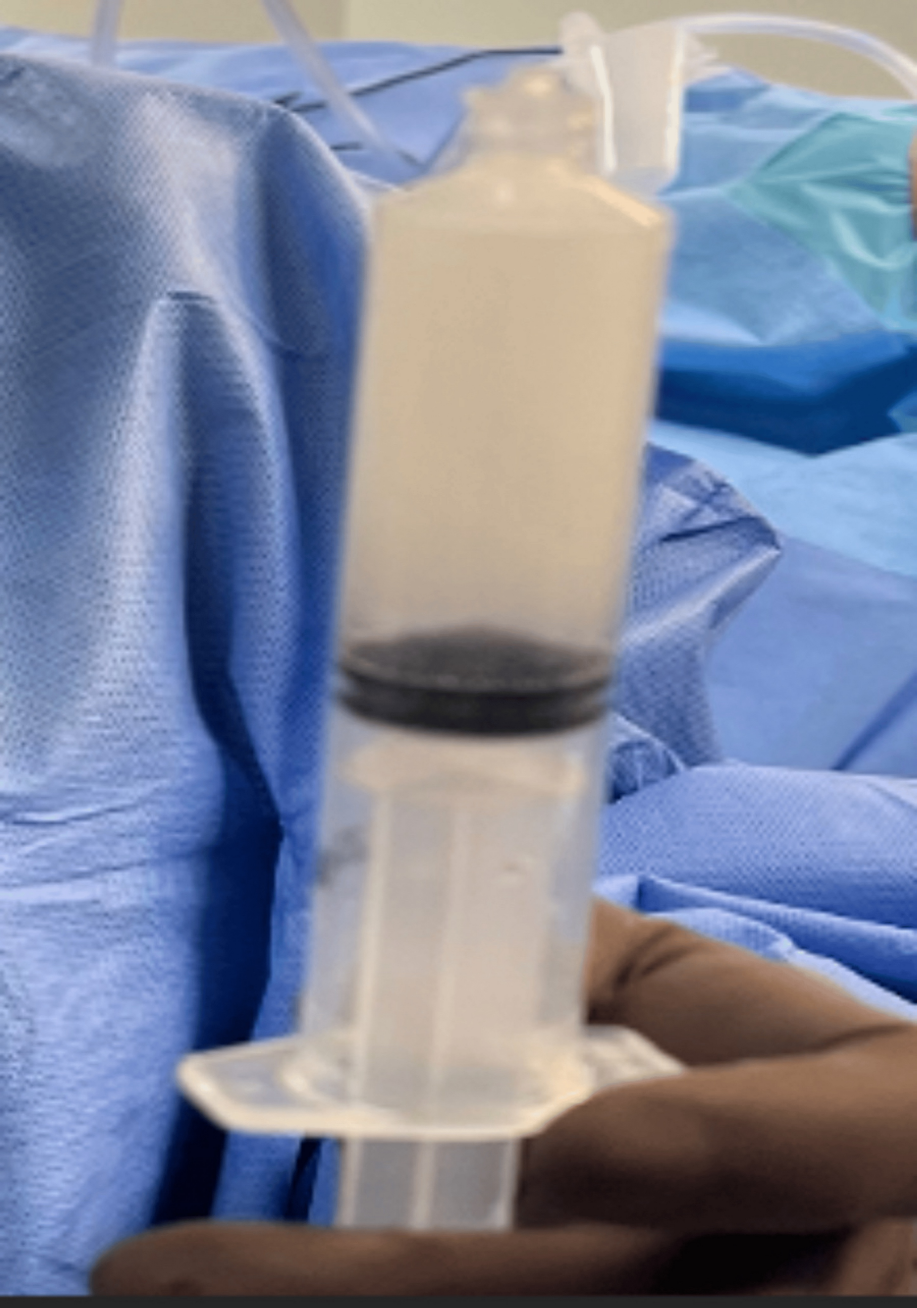
Saline is added until a fluid mixture is obtained.

**Figure 4:**
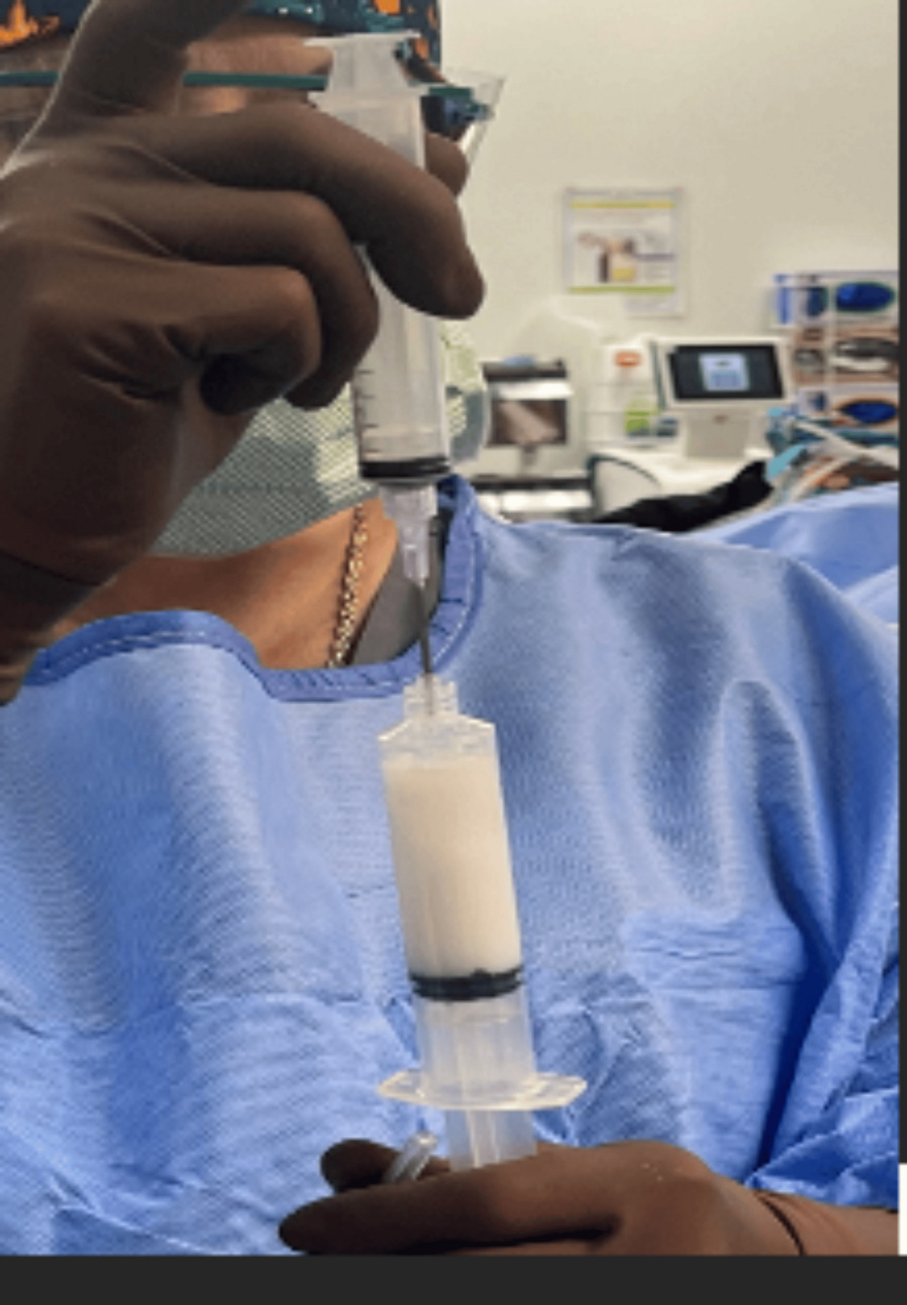
The two mixtures are then combined into the 20 cc syringe to create an injectable mixture.

**Figure 5:**
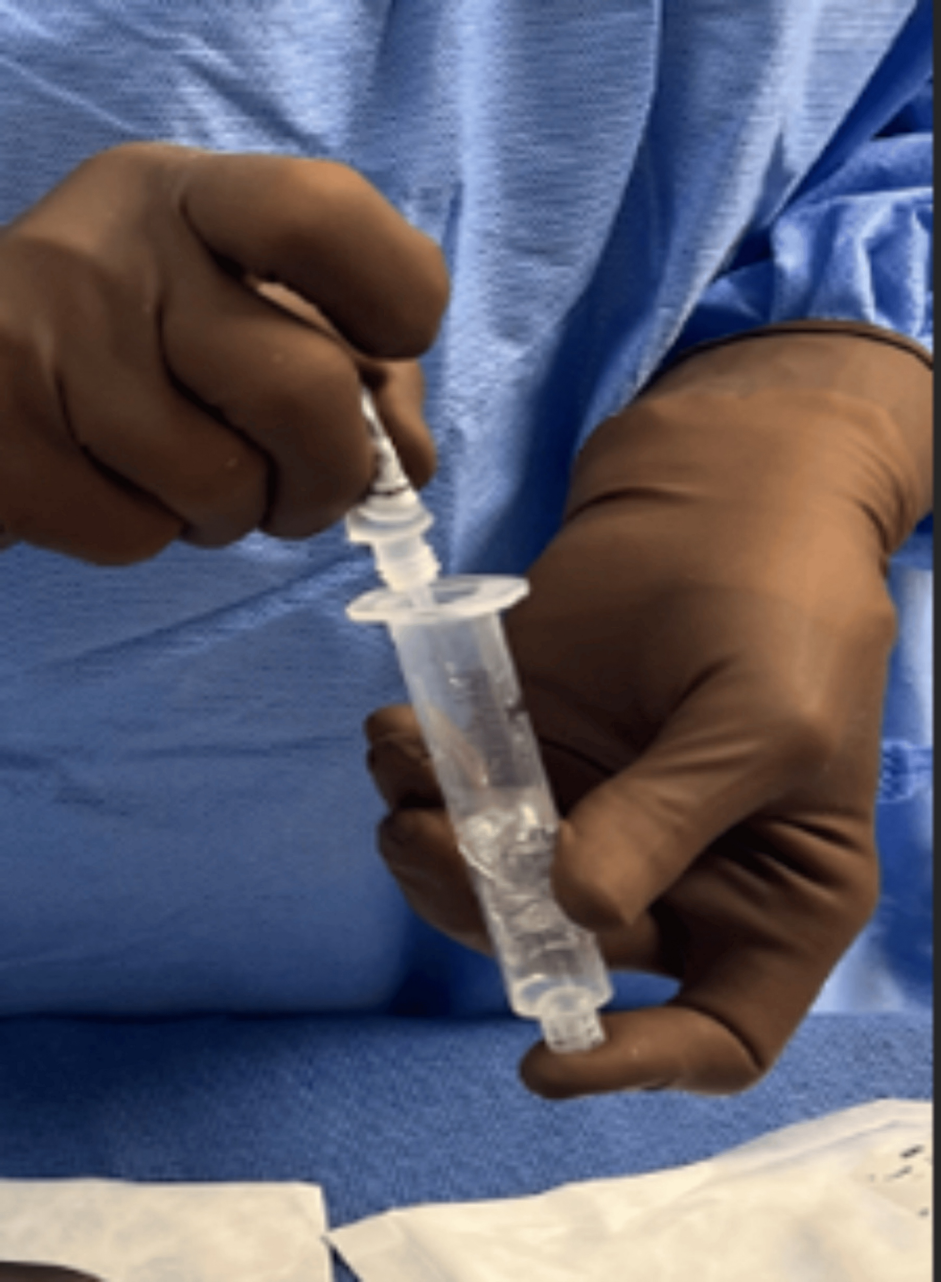
Separately, a hyaluronic acid-impregnated alginate graft is folded and placed in a 10 cc leur lock syringe and 1-2 cc of aqueous citrate is added.

**Figure 6:**
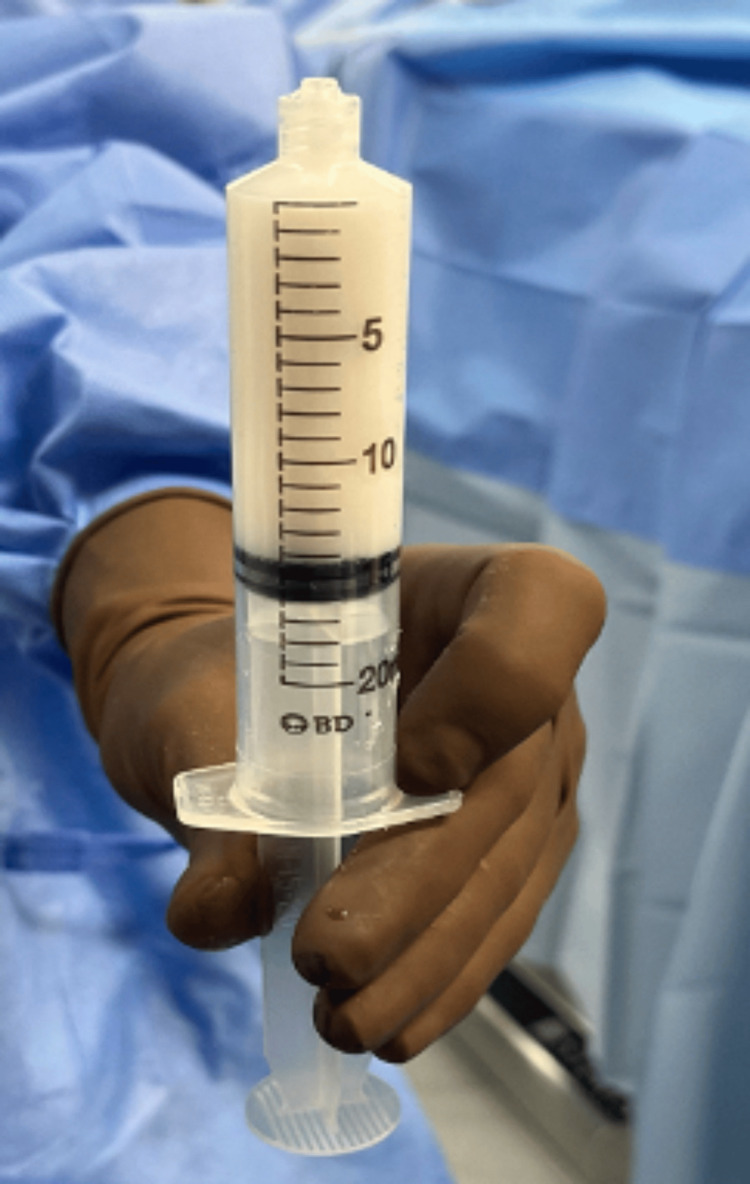
Vancomycin contained within a hyaluronic acid-impregnated alginate carrier.

Vancomycin (1 g) powder is placed in a specimen cup (Figure 1). The powder is transferred into a 20 cc luer lock syringe and 6-8 cc of sterile saline is gradually added while manually mixing with a hemostat/freer elevator (Figure 2). Saturation of the powder improves the initial thick consistency and saline is added until a fluid mixture is obtained (Figure 3). Separately, a hyaluronic acid-impregnated alginate graft (5 by 5 cm, VersaWrap) is folded and placed into a 10cc leur lock syringe and 1-2 cc of aqueous citrate is added and mixed until a clear fluid is observed (Figure 4). The two are then combined into the 20 cc syringe to create an injectable mixture containing vancomycin with a hyaluronic acid-impregnated alginate mixture (Figures 5, 6). A large bore needle is then used for injection.

Modified technique (using prepackaged graft)

**Figure 7:**
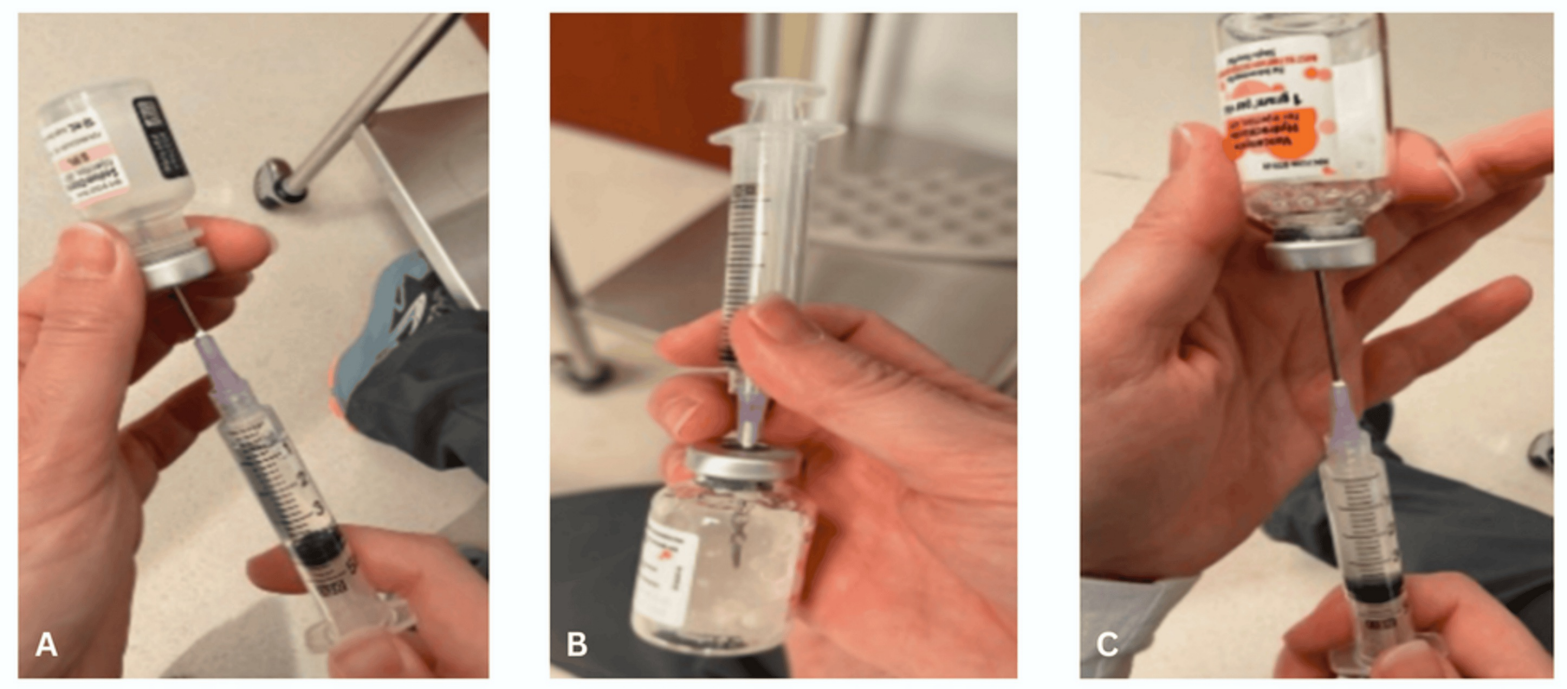
A 10 cc luer lock syringe is used by the circulating nurse to (A) withdraw 4-6 cc of sterile saline. (B) The sterile saline is injected into a self-contained vial of vancomycin (1 g) powder, saturating the powder into a liquid. (C) It is then withdrawn.

**Figure 8:**
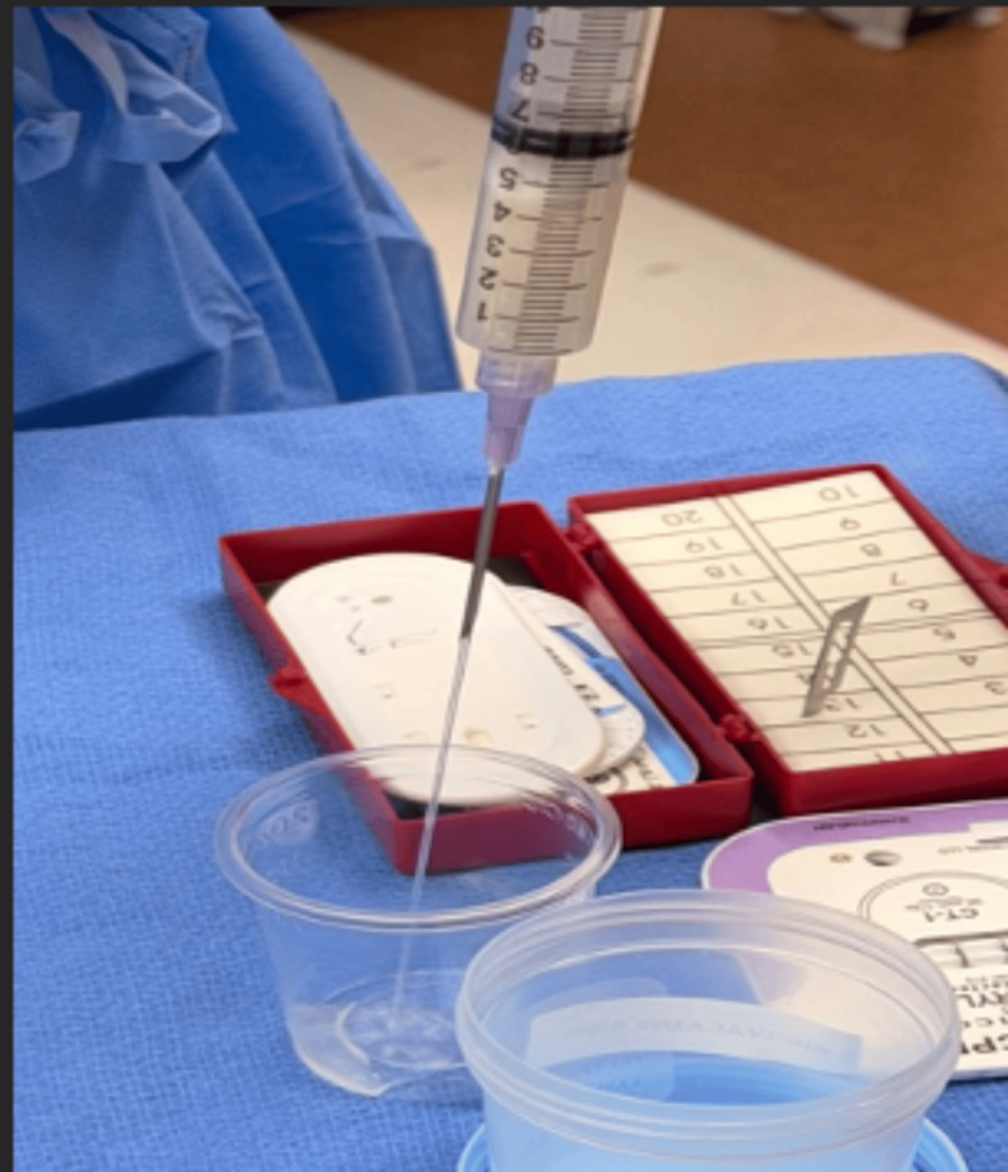
Vancomycin mixed with saline is ejected into a specimen cup on the operating room table.

**Figure 9:**
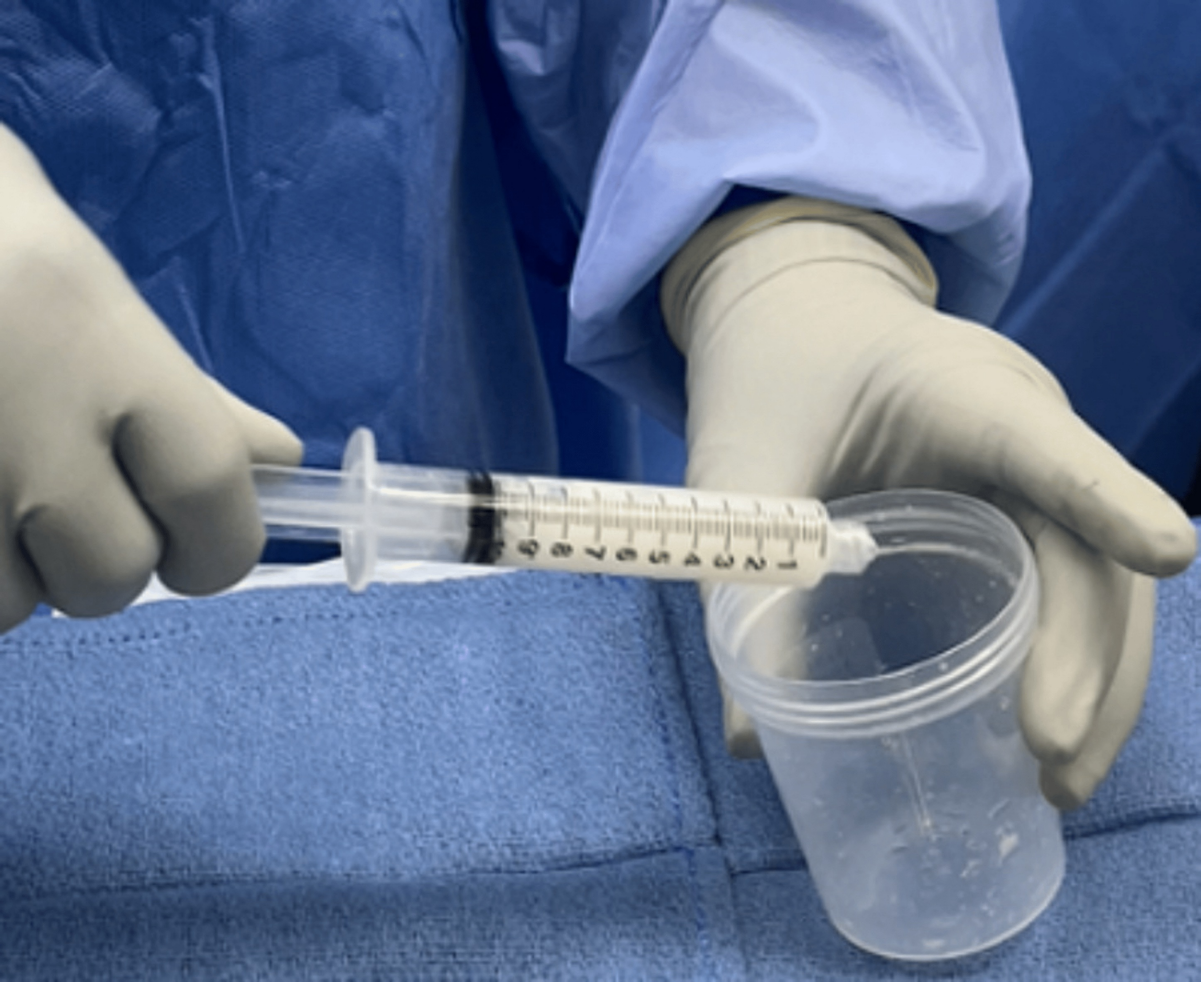
The fluid containing vancomycin from the specimen cup is withdrawn an injectable mixture of vancomycin with a hyaluronic acid-impregnated alginate carrier

**Figure 10:**
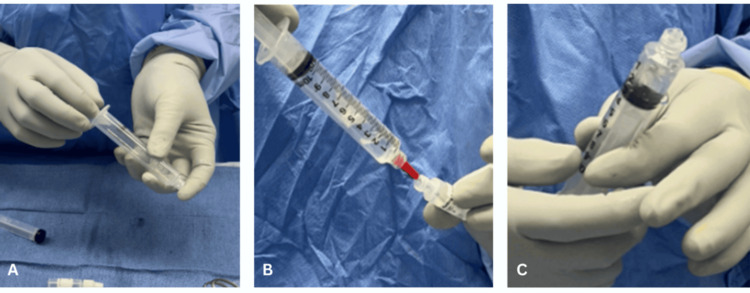
Hyaluronic acid-impregnated alginate graft is (A) folded and placed into a 10 cc leur lock syringe, (B) 1-2 cc of aqueous citrate is withdrawn and (C) mixed.

A 10 cc luer lock syringe is used by the circulating nurse to withdraw 4-6 cc of sterile saline. The sterile saline is subsequently injected into a self-contained vial of vancomycin (1 g) powder, saturating the powder into a liquid that is then withdrawn and ejected into a specimen cup on the operating room table (Figures 7, 8). Separately, a hyaluronic acid-impregnated alginate graft (5 by 5 cm, VersaWrap) is folded and placed in a 10 cc leur lock syringe and 1-2 cc of aqueous citrate is withdrawn and mixed until a clear fluid is observed (Figure 9). Using the same syringe, the fluid containing vancomycin from the specimen cup is withdrawn, creating an injectable mixture of vancomycin with a hyaluronic acid-impregnated alginate carrier (Figure 10). An 18-gauge needle is then used for injection.

Modified technique (using prepackaged flowable hydrogel)

**Figure 11:**
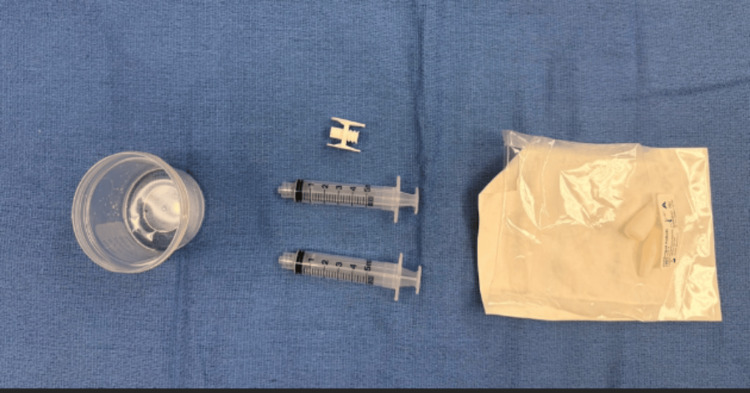
Prepackaged flowable hydrogel kit (Versa-Coat) is opened next to the specimen cup.

**Figure 12:**
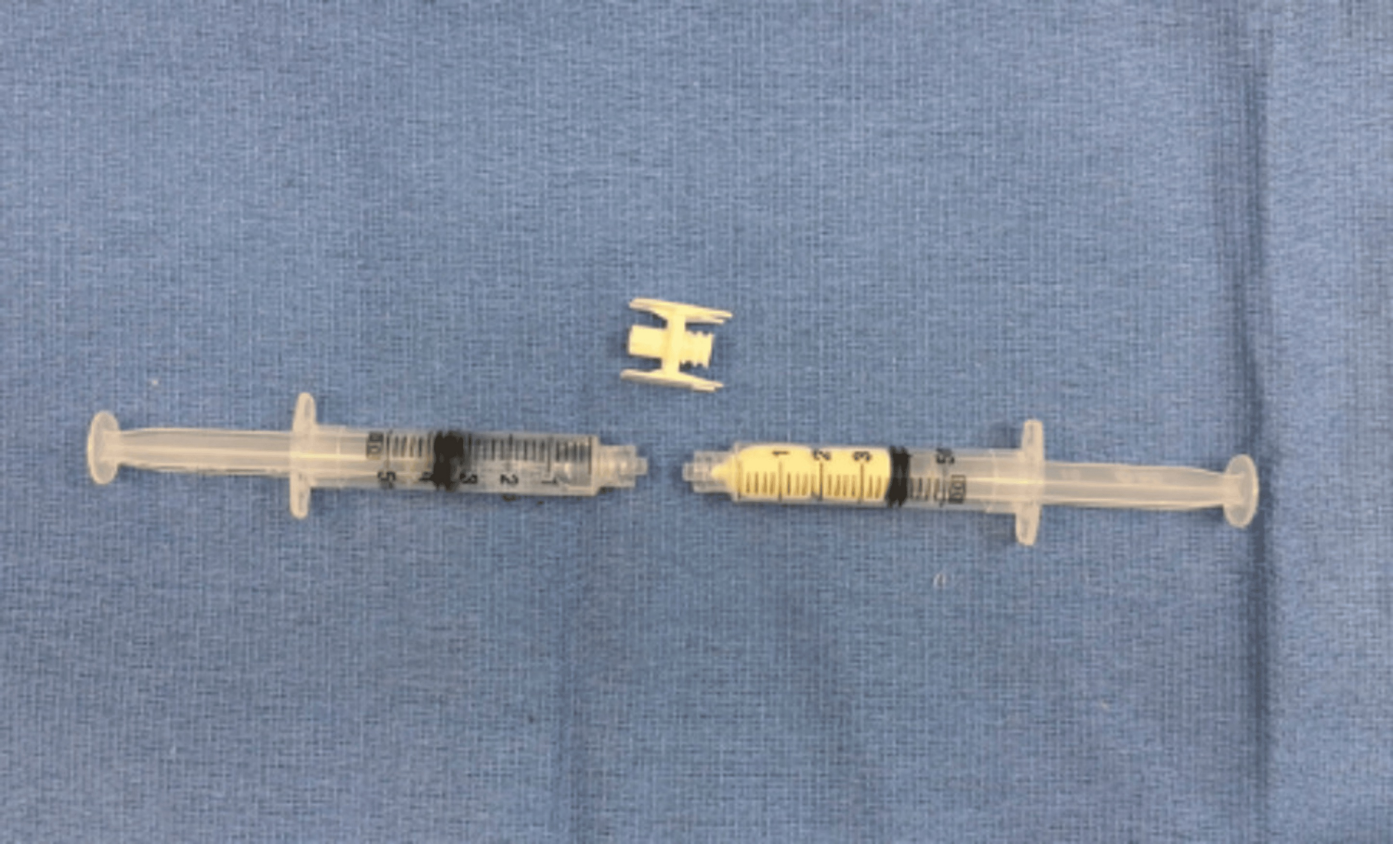
Using one of the syringes, 4 cc of saline containing vancomycin is withdrawn. In the other, 1 cc of citrate is added to two pellets.

**Figure 13:**
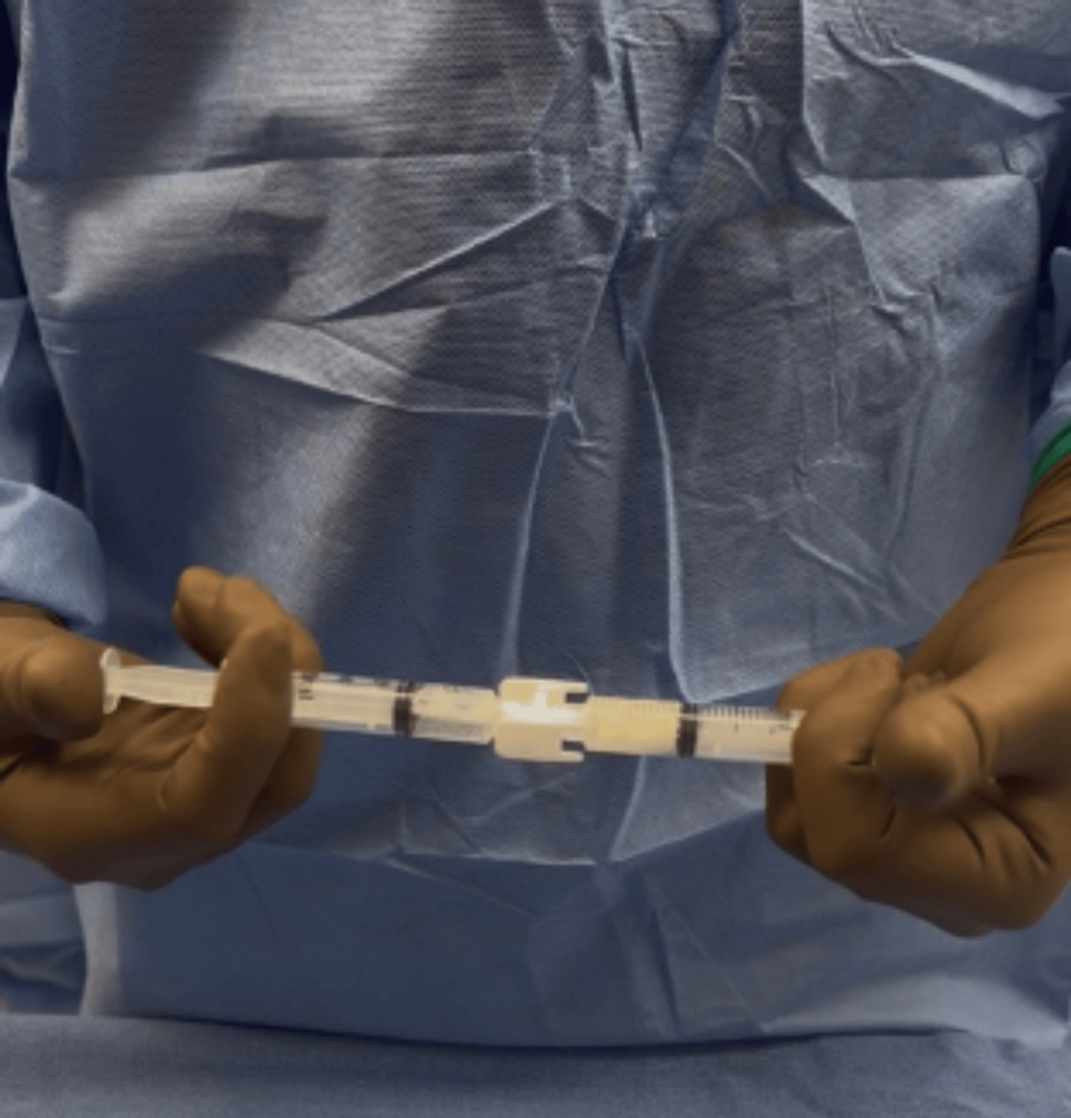
The fluids are manually pushed back and forth between syringes for 30 seconds of mixing, the hyaluronic acid-impregnated alginate pellets dissolve, and the fluid becomes uniform.

**Figure 14:**
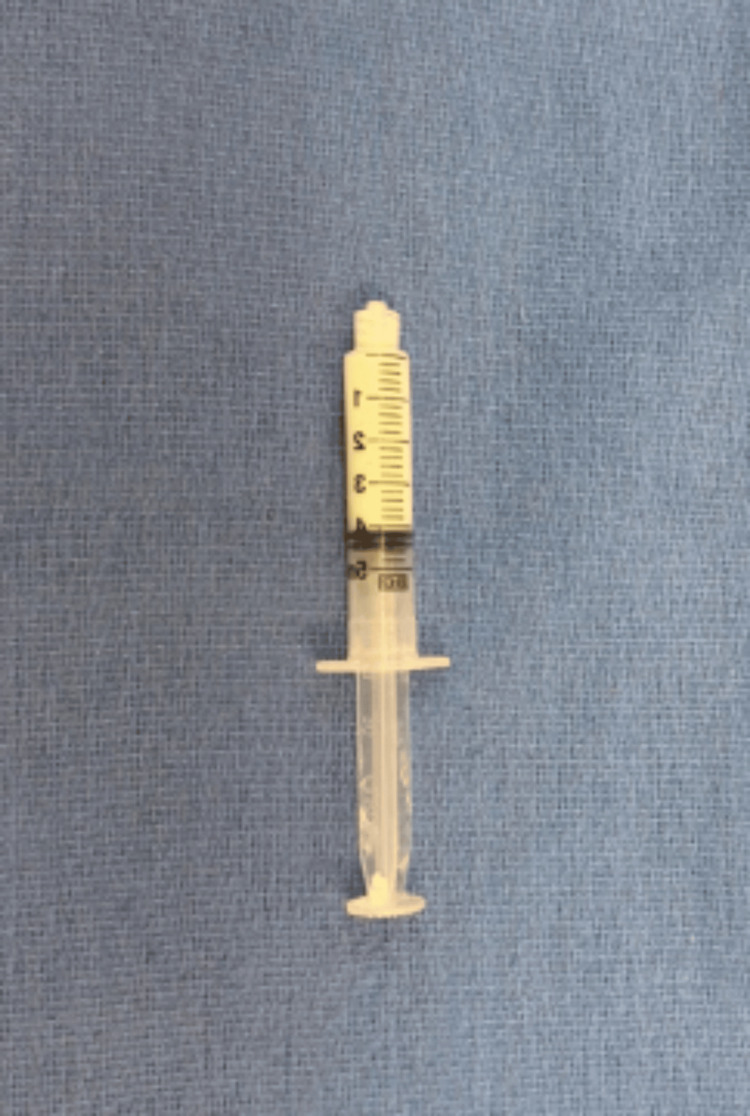
The injectable mixture of vancomycin with a hyaluronic acid-impregnated alginate carrier is then injected using an 18- to 22-gauge needle.

As with the previous technique, a 10 cc luer lock syringe is used to withdraw 4 cc of sterile saline. The saline is injected into a self-contained vial of vancomycin (1 g) powder, and the liquid is then withdrawn and ejected into a specimen cup. On the back table, the prepackaged flowable hydrogel kit (Versa-Coat) is opened next to the specimen cup (Figure 11). Contained within the kit are: two hyaluronic acid-impregnated alginate pellets, aqueous citrate, two 5 cc leur lock syringes, and a syringe connector for mixing. Using one of the syringes, 4 cc of saline containing vancomycin is withdrawn from the specimen cup. In the other, 1 cc of citrate is added to two pellets and the mixing connector is attached (Figure 12). As the fluids are manually pushed back and forth between syringes for 30 seconds of mixing, the hyaluronic acid-impregnated alginate pellets dissolve and the fluid becomes uniform (Figure 13). An 18-22 gauge needle is used for injection (Figure 14).

## Discussion

The purpose of the present article is to describe the author's techniques for injectable vancomycin powder contained within a hyaluronic acid-impregnated alginate carrier for prophylaxis against SSI.

Topically applied vancomycin powder is known to be an effective method to mitigate the risk of SSI, with few adverse reactions and at a nominal cost. However, the optimal dosage, resultant concentration, and duration of activity for topical vancomycin in foot and ankle surgery remains to be determined. As previously discussed, higher concentrations (500 μg/ml, 5000 μg/ml) may impair the viability and osteogenic differentiation of hMSCs [10] and wound drainage and maceration are not uncommon. Clinically relevant doses of vancomycin powder, in contrast, have not been shown to impair osteogenesis or fracture healing in a diabetic rodent fracture model [11]. From the referenced studies and available research, one may surmise that the effect of topical vancomycin on hMSCs is both concentration- and time-dependent. 

The author's rationale for combining vancomycin powder with an impregnated carrier possessing a gradual dissolution profile over time was to: (1) prolong the physiologic levels of the antibiotic locally, improving efficacy; (2) minimize concentration spikes that could potentially affect hMSCs; and (3) reduce postoperative wound drainage. Over the past 12 months, the author has anecdotally observed a reduction in the incidence of both soft tissue and infectious complications, particularly in high-risk populations. The author's modifications to the original technique help optimize workflow, reduce the risk of carrier dissolution by using less saline, and provide a more uniform liquid for injection using 18- and 22-gauge needles. The author recommends the use of either of the modified techniques in place of the original technique for the abovementioned reasons.

## Conclusions

The combination of vancomycin powder with a hyaluronic acid-impregnated alginate carrier for prophylaxis against SSI is a novel technique that may prove superior to topical vancomycin alone. As with any described technique, it is important that surgeons balance enthusiasm with a healthy level of skepticism. Anecdotal observation, after all, is not based on evidence, and innovation is ultimately differentiated from experimentation by objective outcome data. In vitro elution testing, comparative cohort studies, and cost analysis will be salient in discerning the optimal delivery methods for topically applied vancomycin powder.
